# Shift in Bacterial Community Structure Drives Different Atrazine-Degrading Efficiencies

**DOI:** 10.3389/fmicb.2019.00088

**Published:** 2019-01-30

**Authors:** Xiaowei Liu, Kai Chen, Shaochuang Chuang, Xihui Xu, Jiandong Jiang

**Affiliations:** Department of Microbiology, Key Lab of Microbiology for Agricultural Environment, Ministry of Agriculture, College of Life Sciences, Nanjing Agricultural University, Nanjing, China

**Keywords:** atrazine-degrading consortium, community structure, catabolic function, species abundance, degrading efficiency

## Abstract

Compositions of pollutant-catabolic consortia and interactions between community members greatly affect the efficiency of pollutant catabolism. However, the relationships between community structure and efficiency of catabolic function in pollutant-catabolic consortia remain largely unknown. In this study, an original enrichment (AT) capable of degrading atrazine was obtained. And two enrichments – with a better/worse atrazine-degrading efficiency (ATB/ATW) – were derived from the original enrichment AT by continuous sub-enrichment with or without atrazine. Subsequently, an *Arthrobacter* sp. strain, AT5, that was capable of degrading atrazine was isolated from enrichment AT. The bacterial community structures of these three enrichments were investigated using high-throughput sequencing analysis of the 16S rRNA gene. The atrazine-degrading efficiency improved as the abundance of *Arthrobacter* species increased in enrichment ATB. The relative abundance of *Arthrobacter* was positively correlated with those of *Hyphomicrobium* and *Methylophilus*, which enhanced atrazine degradation via promoting the growth of *Arthrobacter*. Furthermore, six genera/families such as *Azospirillum* and *Halomonas* showed a significantly negative correlation with atrazine-degrading efficiency, as they suppressed atrazine degradation directly. These results suggested that atrazine-degrading efficiency was affected by not only the degrader but also some non-degraders in the community. The promotion and suppression of atrazine degradation by *Methylophilus* and *Azospirillum*/*Halomonas*, respectively, were experimentally validated *in vitro*, showing that shifts in both the composition and abundance in consortia can drive the change in the efficiency of catabolic function. This study provides valuable information for designing enhanced bioremediation strategies.

## Introduction

Atrazine is one of the most widely used *S*-triazine herbicides, and approximately 70,000–90,000 tons of atrazine are used worldwide annually. Because of its low adsorption and long half-life in soil, atrazine residue in soil poses a great risk of pollution for surface water and groundwater. For example, the concentrations of atrazine detected in groundwater frequently exceed the maximum level for drinking water, making it a potential threat to ecosystems and human health (Jablonowski et al., 2011). The dissipation of atrazine in the environment is mainly attributed to its decomposition by microbial catabolism (Huang et al., 2016). Bacterial strains that can utilize atrazine as the sole source of carbon and nitrogen have been isolated since 1995 (Huang et al., 2016), and most of these isolates belong to the genus *Arthrobacter* (Sajjaphan et al., 2004; Xie et al., 2013; Wang et al., 2016). Though the activity of atrazine-degraders is important for the bioremediation of atrazine-contaminated sites, it is usually difficult for these degraders to survive in exogenous niches. The poor survival of inoculated degraders in soil is usually due to competition from indigenous microorganisms, which might influence or even lead to the elimination of the inoculated degraders. Moreover, the characteristics of the soil environment, including soil properties, water content, and available energy substances, are also important factors for the survival of inoculated degraders (Nannipieri et al., 2003).

In natural environments, the catabolism of organic pollutants by microbes is rarely performed by a single species; it is usually carried out by complementary or overlapping degradation steps catalyzed by microbial consortia (Jeon and Madsen, 2013). On the other hand, syntrophy–where the growth of one strain is dependent on the nutrients, growth factors, or substrates provided by other symbiotic strains–is also important for the catabolism of pollutants (Chen et al., 2013). Synergistic catabolism by various bacterial communities has been reported for the degradation of different pollutants, such as dicamba, *alpha*-hexachlorocyclohexane, fluorobenzene, linuron, and 4-chloroaniline (Taraban et al., 1993; Manonmani et al., 2000; Carvalho et al., 2002; Dejonghe et al., 2003). Similarly, many studies have shown that microbial consortia play important roles in the degradation and mineralization of atrazine (Souza et al., 1998; Smith et al., 2005; Dehghani et al., 2007; Yang et al., 2010). For instance, in a four-strain atrazine-degrading consortium, *Clavibacter michiganense* strain ATZ1 provided ring opening products by removing the side chain of atrazine for *Pseudomonas* sp. strain CN1 (Souza et al., 1998). In an eight-strain microbial consortium, a *Nocardioides* sp. strain dechlorinated atrazine, and the other seven strains utilized the downstream products (Smith et al., 2005). Another bacterial consortium, composed of seven gram-negative bacteria and one gram-positive bacterium, was able to use atrazine as the sole nitrogen source for growth (Dehghani et al., 2007). In addition, *Klebsiella* sp. A1 and *Comamonas* sp. A2 were reported to catabolize atrazine synergistically (Yang et al., 2010). Therefore, synergistic cooperation for metabolism exists universally in microbial communities, and in many cases, it will enhance the degradation of complex pollutants (Horemans et al., 2013).

Interactions between community members are dynamic and complex (Xu X. H. et al., 2018). The microbial community composition and relationship between their members not only affect the catabolic efficiency of pollutants but also determine the final fate of pollutants (Kato et al., 2005). However, the relationships between the catabolic function and structure of the microbial community have not been extensively investigated (Fang et al., 2015; Bazhanov et al., 2016).

With the development of high-through sequencing technology, amplicon pyrosequencing of the 16S rRNA V3–V4 hypervariable regions provides a powerful tool to comprehensively define the bacterial composition, abundance, and community structure of microbial consortia. By analyzing the features of the microbial community structure of samples with different degrading efficiencies, it is possible to determine the relationships between the key degraders and other indirect non-degraders, as well as the effect of supporting species on the degrading efficiencies. Compared to the complex and varied microbial community in soils, the simple and stable consortia in enrichments obtained from soils make it much easier to focus on determining the relationships between the catabolic function and microbial community structure. In this study, three enrichments with different atrazine-degrading efficiencies were obtained, and the bacterial community structures of these three enrichments were analyzed. The effects of the shift in bacterial community structure on the atrazine-degrading efficiencies were clarified *in silico* and experimentally tested *in vitro*. Our findings will expand the understanding of the roles of bacterial community structure in the catabolism of pollutants and provide guidance for the design of efficient microbial consortia for the enhanced bioremediation of pollutant-contaminated sites.

## Materials and Methods

### Soil, Reagents, and Media

The atrazine-contaminated soil sample for enrichment was collected from an atrazine-producing pesticide factory in Boxing (37°12′ N, 118°12′ E), Shandong, China. Atrazine (>97% purity) was purchased from Aladdin Industrial Corporation (Shanghai, China). Mineral salt medium (MSM) (Kumar and Singh, 2016) supplemented with atrazine and Luria-Bertani medium (LB) were used for degradation assays and cell culture, respectively.

### Obtaining the Original Enrichment With Atrazine-Degrading Ability and Isolation of an Atrazine-Degrading Strain

Five grams of the selected atrazine-contaminated soil was added to 100 ml MSM containing 50 mg/l atrazine and enriched at 30°C for 10 days. After two rounds of enrichment, an original enrichment (AT) with atrazine-degrading ability was obtained. The atrazine-degrading ability was assessed by detecting the remaining concentration of atrazine by high-performance liquid chromatography (HPLC) and comparing to that of the controls. The sample was filtered through a 0.22-μm Millipore membrane, and the atrazine concentration was determined using UltiMate^®^ 3000 Titanium System HPLC (Thermo Fisher Scientific) equipped with a VWD-3100 Single Wavelength Detector; the detection wavelength was 243 nm. Samples (10 μl) were separated on a C18 reversed-phase column (4.6 × 250 mm, 5 μm; Agilent Technologies); the column temperature was set at 30°C, and the mobile phase consisted of methanol and water at a ratio of 70:30 (vol/vol) flowing at 0.8 ml/min.

To isolate strains capable of degrading atrazine, the original enrichment AT was serially diluted and directly spread onto MSM agar containing 100 mg/l atrazine and incubated at 30°C for 3–5 days. An individual colony with a transparent halo was selected, and its atrazine-degrading ability was further determined by HPLC as described above. The isolated strain was identified based on its morphological, physiological, and biochemical properties as well as the 16S rRNA gene sequence analysis (Yang et al., 2014).

### Obtaining Enrichments With Different Atrazine-Degrading Efficiencies

The enrichments with better and worse atrazine-degrading efficiencies (ATB and ATW) were derived from AT by incubated it with and without atrazine, respectively. In detail, the original enrichment AT was divided evenly into two subcultures and further enriched with or without 50 mg/l atrazine. After two rounds of enrichment (20 days), the atrazine-degrading efficiencies of the two sub-enrichments and the original enrichment AT were analyzed and compared. The OD_600_ of each enrichment was adjusted to the same value (OD_600_ = 0.2) when assessing its degrading efficiency. Finally, one enrichment with a better atrazine-degrading efficiency (named ATB) and one enrichment with a worse atrazine-degrading efficiency (named ATW) were derived from the original enrichment AT.

### 16S rRNA Amplicon Pyrosequencing

The genomic DNA of the enrichments was extracted using the FastDNA SPIN Kit (MP Biomedicals, Santa Ana, United States) and determined with a NanoDrop ND-1000 spectrophotometer (Thermo Fisher Scientific, Waltham, MA, United States). The bacterial or archaeal 16S rRNA fragment covering the V3 and V4 variable regions was amplified with the primer pair 338F (5′-actcctacgggaggcagca-3′)/806R (5′-ggactachvgggtwtctaat-3′) (Zakrzewski et al., 2012; Klindworth et al., 2013). Unique 5–8 bp barcodes (Supplementary Table S1) for different samples were incorporated into the primers for multiplex sequencing (Parameswaran et al., 2007). The PCR system and amplification conditions were similar to those described by Mao et al. (2015). The PCR products were quantified with a QuantiFluor-ST blue fluorescence quantitative system (Promega, United States). The amplicon library was paired-end sequenced (2 × 300) on an Illumina MiSeq platform according to the standard protocols (Shanghai BIOZERON Co., Ltd., China). The raw sequencing data were deposited in the National Center for Biotechnology Information (NCBI) Sequence Read Archive (SRA) under the accession number PRJNA498798.

### Sequence Analysis

Paired-end reads were assigned to each sample according to the unique barcodes and merged into long reads according to the overlap relationships using FLASH (version 1.2.7) (Magoc and Salzberg, 2011). Low-quality merged reads were filtered using QIIME (version 1.17) (Caporaso et al., 2010). The filtered 16S rRNA sequences were clustered into operational taxonomic units (OTUs) using UPARSE version 7.1 (Edgar, 2013). Taxonomic analysis at a 97% sequence identity of the representative sequence was carried out using the RDP classifier Bayesian algorithm and comparison with the species in the 16S Database (Desantis et al., 2006).

Single sample diversity analysis (Alpha diversity), including a series of statistical analysis indices, was used to estimate the species richness and diversity of the community. The rarefaction analysis based on Mothur (version v.1.30.1) was conducted to reveal the diversity indices, including the Chao1, Coverage, Simpson, and Shannon diversity indices, at 97% identity (Schloss et al., 2011; Paul et al., 2016). Beta diversity was analyzed to investigate the similarity of bacterial community structure among groups. The distance matrix was calculated using the Bray Curtis algorithm with QIIME, and the principal component analysis (PCA) (Wang et al., 2012) was performed using the ‘vegan’ package with the R language (Oksanen et al., 2016). LEfSe (version 1.0) was used to detect differentially abundant genera in the three groups for biomarker discovery (Segata et al., 2011), and the threshold on the logarithmic linear discriminant analysis (LDA) score for discriminative features was set to 4.0. Pearson’s correlation coefficients between the atrazine degradation rate and the abundance of each genus and among abundances of all genera were analyzed using the ‘cor’ function with the R language. And robust correlations in Pearson’s correlation test were assessed based on probability values using the ‘corr. test’ function of the ‘psych’ package with the R language (*p*-value < 0.05). The *p*-values were adjusted with “fdr” methods to control the false discovery rate using the ‘corr. test’ function. PICRUSt software (version 1.0.0) was used to estimate the composition of functional genes in samples (Parks et al., 2014). These predictions were pre-calculated for genes in the Kyoto Encyclopedia of Genes and Genomes (KEGG) catalog (Ogata et al., 1999).

### Degradation of Atrazine by Different Consortia

*Methylophilus* sp. SP1, *Halomonas* sp. N8, and *Azospirillum* sp. A1 (stocked in our laboratory) were used to experimentally test the effects of these non-degraders on the atrazine-degrading efficiency of *Arthrobacter* sp. AT5 (isolated from this study). Atrazine degradation carried out by one-strain (strain AT5) and two-member consortia (combination of strain SP1, N8, or A1 with strain AT5, respectively) was measured. *In vitro* experiments, we used different initial concentrations of members in the consortia, including 1:1, 3:1, and 1:5 for strain AT5 to strain SP1/N8/A1. All strains were grown in LB medium at 30°C. To measure atrazine degradation, a single strain or consortia were grown in 20 ml MSM medium containing 50 mg/l atrazine for 72 h at 30°C. The concentration of atrazine in each culture was detected every 12 h by HPLC as described above. All treatments were carried out in triplicate.

## Results

### Enrichments With Different Atrazine-Degrading Efficiencies and Isolation of an Atrazine-Degrading Strain

**FIGURE 1:**
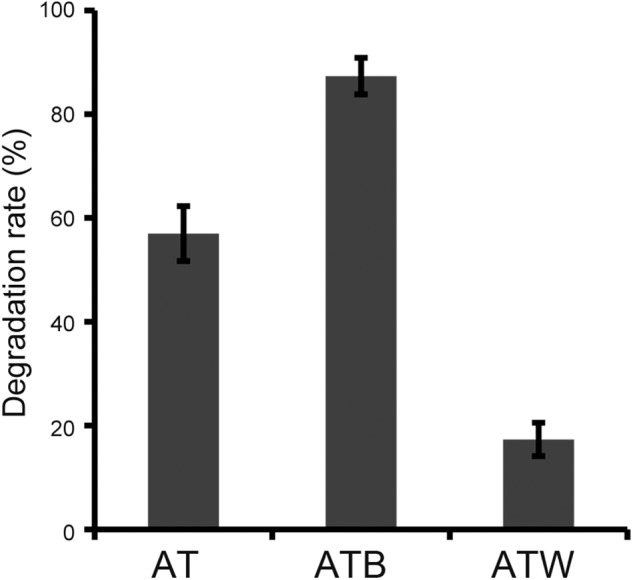
Degradation rates of 50 mg/l atrazine at 48 h by three enrichments at the same inoculum concentration. The data are the average of three replicates.

An original enrichment (AT) with atrazine-degrading ability was obtained by the enrichment of atrazine-contaminated soil collected from a factory in Shandong, China. A bacterial strain, AT5, that could use atrazine as the sole nitrogen and carbon source energy for growth was isolated from enrichment AT by the direct dilute-plating method, and strain AT5 completely removed 50 mg/l atrazine in 4 days. The 16S rRNA gene sequence of strain AT5 showed 99% similarity to that of *Arthrobacter ureafaciens*. According to the phylogenetic tree of the 16S rRNA gene, strain AT5 was finally identified as an *Arthrobacter* species (Supplementary Figure S1). Two sub-enrichments (ATB and ATW) were derived from the original enrichment AT, and the degrading efficiencies of these three enrichments were compared, showing that enrichments AT, ATB, and ATW degraded 57.0%, 87.3%, and 17.3% of 50 mg/l atrazine in 48 h (Figure 1).

### Pyrosequencing of the 16S rRNA Amplicon of the Three Enrichments

A total of 505,863 valid sequences and 470,625 high-quality sequences were generated from nine samples (each enrichment with three replicates), with an average of 52,292 sequences per sample. The average sequence length was 440.57 bp. Clustering of all high-quality sequences at 97% identity resulted in 867 OTUs for the nine samples (Supplementary Table S2). The number of OTUs varied greatly between the three enrichments (ranging from 94 to 791), and more OTUs were found in enrichment AT than in enrichments ATB and ATW.

### Diversity Analysis and Community Structure

The results of the alpha diversity indices of Chao1, Shannon, and Simpson are shown in Table 1. The Chao1 richness index of AT (801) was much higher than those of ATB (116) and ATW (156), indicating that AT had a higher bacterial abundance. The Shannon diversity index of AT (5.32) was higher than those of ATB (1.46) and ATW (2.86) (Table 1). In addition, the Simpson diversity index of AT (0.0135) was the lowest among the three enrichments, demonstrating that AT had the highest bacterial diversity. Good’s coverage estimator for each sample was over 99%, indicating that the sequencing depth was sufficient to saturate the bacterial diversity.

**Table 1:** Alpha diversity indices at a 97% identity threshold. Values are the mean followed by standard error. Different superscripts (a, b, and c) indicate statistically significant differences (*p* < 0.05) according to the Tukey test.

Sample	Chao1	Coverage	Shannon	Simpson
AT	800.7 ± 3.8^a^	0.9996	5.31 ± 0.03^a^	0.0135^c^
ATB	116 ± 9.5^c^	0.9996	1.46 ± 0.05^c^	0.3684^a^
ATW	156 ± 11.4^b^	0.9997	2.86 ± 0.12^b^	0.1271^b^

**FIGURE 2:**
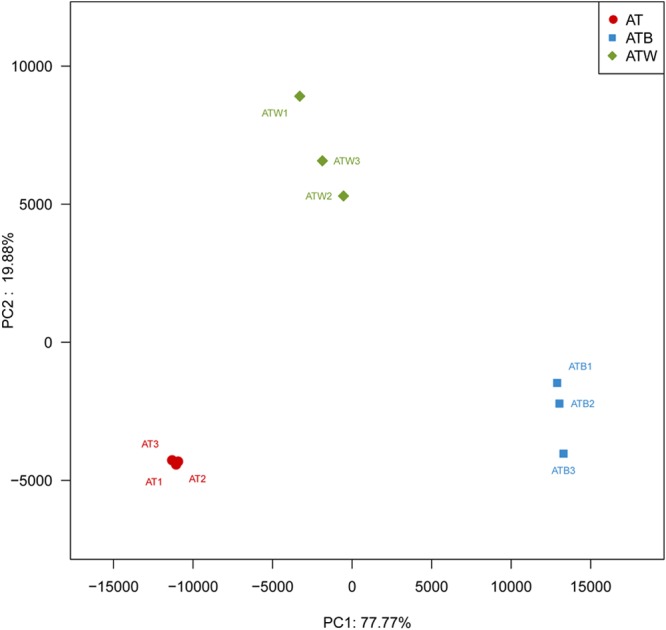
PCA analysis of multiple samples. Each sample is represented by a dot. PC1 explained 77.77% of the variation observed, and PC2 explained 19.88% of the variation.

**FIGURE 3:**
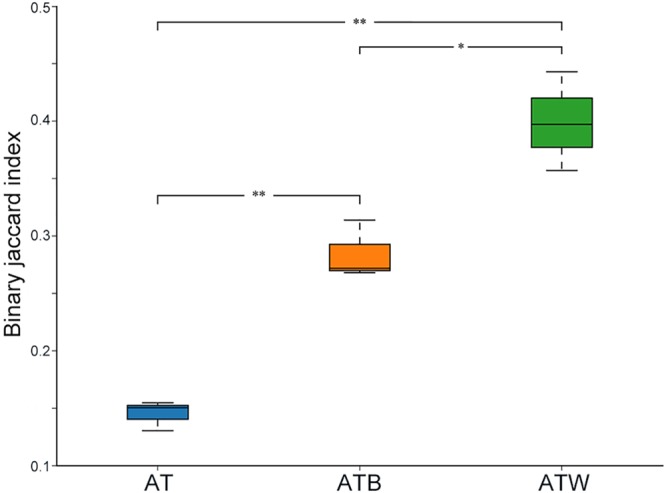
Inter-group beta diversity box diagram. The x-axis represents the grouping, the y-axis represents the distance, and the boxes of different colors represent each group. ^∗^*p* < 0.05; ^∗∗^*p* < 0.01.

Figure 2 shows the PCA results based on the genus level classification. Clear segregations in community structures among the three enrichments were detected, with the first two principal components representing 77.77% and 19.88% of the total variations. These results indicated obviously different community structures among the three types of enrichments, while the species composition in each type of enrichment was very similar. Similar results were found based on a hierarchical clustering tree (Supplementary Figure S2). Consistently, significant differences in species beta diversity between AT and ATB (Jaccard difference index: 0.887) and between AT and ATW (Jaccard difference index: 0.869) were detected (Figure 3).

### Bacterial Abundance and Differential Microbial Compositions

**FIGURE 4:**
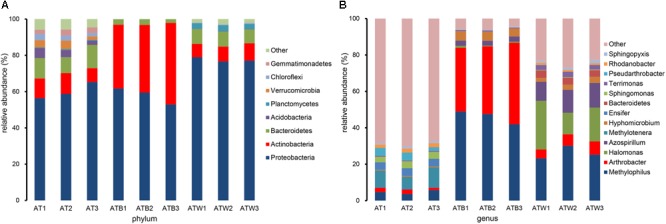
The abundance percentages of the bacterial phyla **(A)** and genera **(B)**. The predominant bacteria with a relative abundance >1% are shown at the phylum and genus level, and the rest are merged into the other.

In all samples, a total of 26 phyla, and 362 genera were detected. The taxonomic distributions of the predominant bacteria (relative abundance >1%) at different levels of the three enrichments are shown in Supplementary Figure S3. Clear differences in components at the phylum and genus levels between different enrichments were detected (Figure 4). Twenty-five phyla in enrichment AT were detected, and the 9 most abundant phyla were *Proteobacteria, Bacteroidetes, Actinobacteria, Acidobacteria, Verrucomicrobia, Gemmatimonadetes, Chloroflexi, Saccharibacteria*, and *Cyanobacteria*, together accounting for 96.48% of the total sequences. There were only 9 phyla in enrichment ATB, and the 3 phyla *Proteobacteria, Actinobacteria*, and *Bacteroidetes* accounted for 99.87% of the total sequences. The enrichment ATW had 16 phyla, including the 5 most abundant phyla *Proteobacteria, Actinobacteria, Bacteroidetes, Planctomycetes*, and *Chlorobi*, together accounting for 98.48% of the total sequences. Compared with enrichment AT, 10 phyla, including *Verrucomicrobia*, disappeared in enrichments ATB and ATW.

**FIGURE 5:**
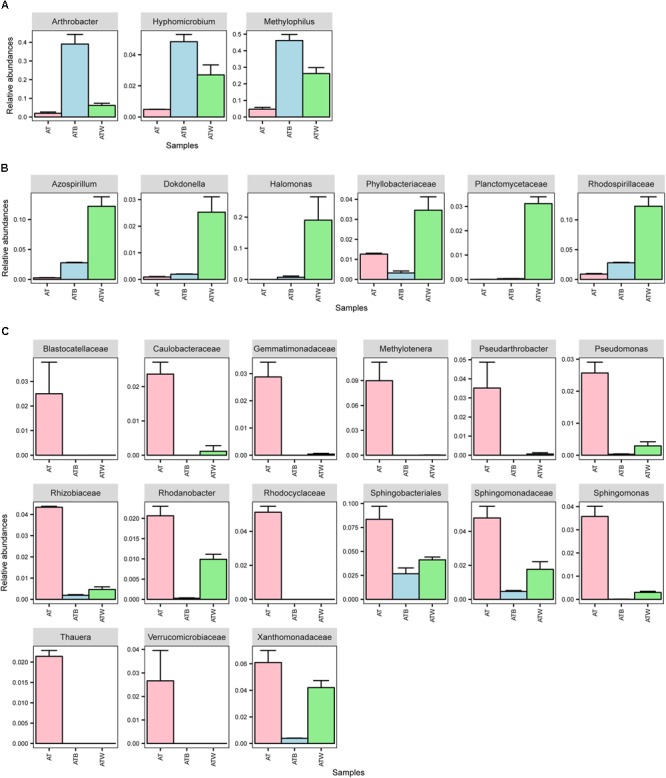
Relative abundance of differentially abundant families and genera detected by LEfSe analysis. **(A)** bacteria in group ATB; **(B)** bacteria in group ATW; and **(C)** bacteria in group AT.

At the genus level, 318, 84, and 159 genera in enrichments AT, ATB, and ATW were detected, respectively. LEfSe analysis was used to identify the species with significant differences (biomarker) in abundance in the three enrichments. In total, 57 potential biomarkers at all levels were detected (Supplementary Figure S4). At the genus level, three potential biomarkers, *Arthrobacter, Hyphomicrobium*, and *Methylophilus*, were found in enrichment ATB (Figure 5), all of which showed the highest abundance in ATB. Three biomarkers, *Halomonas, Azospirillum*, and *Dokdonella*, with significant differences were detected in enrichment ATW, and their abundance increased significantly in ATW compared to those in AT and ATB. The enrichment AT had 7 potential biomarkers, such as *Methylotenera* and *Sphingomonas.* The abundance of the 7 biomarkers showed significantly higher abundance in AT than in the two other enrichments. Particularly, the relative abundance of the genus *Arthrobacter* (involved in the degradation of atrazine) was much higher in enrichment ATB (39.01%) than that in enrichments AT (1.95%) and ATW (6.17%).

## Correlation of Atrazine Degradation Rate and Bacterial Abundances

**FIGURE 6:**
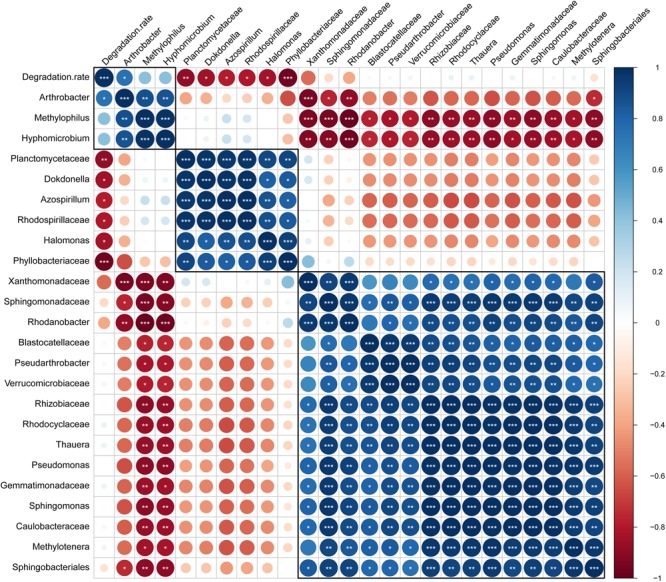
Correlation of atrazine degradation rate and bacterial abundance. Positive and negative correlations are displayed in blue and red colors, respectively. In the correlogram, the circle size and color intensity stand for correlation coefficients. The legend color shows the correlation coefficients and the corresponding colors. The number of stars represents probability values in the pearson’s correlation test. ^∗^*p* < 0.05; ^∗∗^*p* < 0.01; ^∗∗∗^*p* < 0.001.

To identify the bacteria that influenced the efficiency of atrazine degradation, the correlations of the abundance of biomarkers (at the genus and family levels) detected by LEfSe analysis and atrazine degradation rate were analyzed (Figure 6). Only one genus, *Arthrobacter*, from the biomarkers of enrichment ATB, significantly positively correlated with the atrazine degradation rate. However, the two other biomarkers *Hyphomicrobium* and *Methylophilus* showed a significant positive co-occurrence with *Arthrobacter*. These results indicated that biomarkers in enrichment ATB might promote atrazine degradation efficiency directly or indirectly. All the biomarkers in ATW, including *Halomonas, Azospirillum, Dokdonella*, Planctomycetaceae, Rhodospirillaceae, and Phyllobacteriaceae, showed a significant negative correlation with the atrazine degradation rate. Meanwhile, a significant co-occurrence among these bacteria was detected, while no co-occurrence of them with other biomarkers was found. Interestingly, no significant correlation between the 15 biomarkers in enrichment AT and atrazine degradation rate was detected. However, these biomarkers showed a significant negative co-occurrence with the potential atrazine-degradation-enhanced bacteria (biomarkers in enrichment ATW).

**FIGURE 7:**
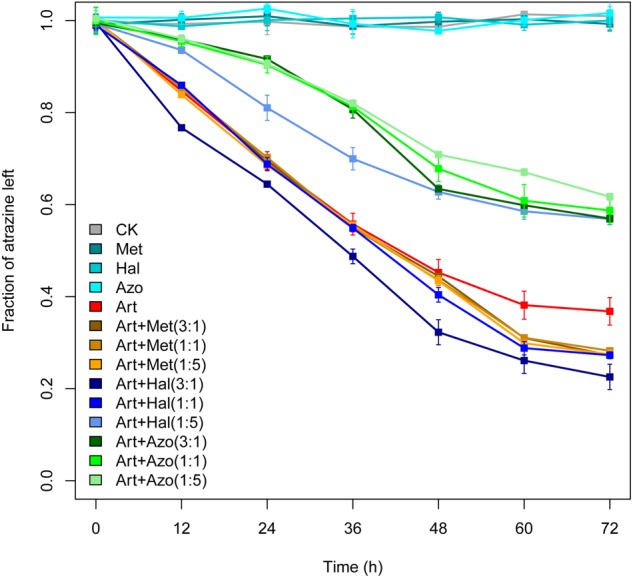
Experimental validations of atrazine degradation by *Arthrobacter* only and by two- member consortia. CK, control; Art, *Arthrobacte*r; Met, *Methylophilus*; Hal, *Halomonas*; Azo, *Azospirillum*. The numbers in brackets represent the ratio of relative abundance of *Arthrobacter* to those of *Methylophilus, Halomonas*, or *Azospirillum* in the test. The strains *Arthrobacter* sp. AT5, *Methylophilus* sp. SP1, *Halomonas* sp. N8, and *Azospirillum* sp. A1 were used. Bars represent the standard errors of the three replicates.

The improvement or suppression of atrazine degradation by the biomarkers in enrichment ATB or ATW was experimentally tested (Figure 7). The relative abundance of the members in the consortia was also analyzed using different concentration ratios of *Arthrobacter* to *Methylophilus, Halomonas*, or *Azospirillum* in the test (1:1, 3:1, and 1:5). The combination of *Arthrobacter* and *Methylophilus* showed a significantly higher atrazine degradation rate than that of *Arthrobacter* only after 48 h of inoculation, and the ratio of relative abundance of *Arthrobacter* to *Methylophilus* did not affect the improvement in the atrazine degradation rate. The combination of *Arthrobacter* and *Azospirillum* inhibited the atrazine degradation rate at all concentrations of *Arthrobacter* and *Azospirillum* used. For consortia with *Arthrobacter* and *Halomonas*, atrazine degradation was suppressed when the abundance of *Arthrobacter* was lower than that of *Halomonas* (1:5). Interestingly, when *Arthrobacter* was combined with a lower abundance of *Halomonas* (3:1), the atrazine-degrading efficiency was significantly improved. Consortia with equal amounts of *Arthrobacter* and *Halomonas* showed improvements in the atrazine-degrading efficiency after 48 h of inoculation compared to that with *Arthrobacter* only. These results revealed that there is a direct relationship between atrazine-degrading efficiency and relative abundance of bacteria in consortia.

### Functionality Analysis

To predict the bacterial functions of the three enrichments, PICRUSt analysis was performed based on the 16S rRNA composition data of each enrichment, and the KEGG database was used to match the chosen reference OTUs (Supplementary Figure S5). Compared with that in enrichment AT, the abundance of genes related to membrane transport was significantly higher in enrichment ATW (Supplementary Figure S5). A significantly higher abundance of genes involved in carbohydrate metabolism and energy metabolism was found in enrichment ATB than that in enrichment AT. In addition, metabolic functions such as membrane transport, cell motility, and energy metabolism were significantly different between enrichments ATB and ATW.

## Discussion

The pollution of soil and water by herbicides, including atrazine, is persistent worldwide, and bioremediation by microorganisms has always been a popular research topic because of its safety and efficiency (Lima et al., 2009; Douglass et al., 2016; Cycon et al., 2017; Xu X. et al., 2018). Many pollutant-degrading strains have been isolated and used for the bioremediation of atrazine-contaminated sites (Devers et al., 2007). However, some supporting species that are not directly related to pollutant biodegradation also play important roles in the efficient catabolism of the pollutants by maintaining the stability of the community structure and/or cross-feeding of the focal degraders (Smith et al., 2005). Therefore, studying the relationships between the microbial community structure and its catabolic function could provide valuable information to promote the development of bioremediation solutions. In this study, the bacterial community structures of three enrichments with different atrazine degradation efficiencies were analyzed, and the relationships between the community structure and atrazine catabolic function efficiency were assessed.

Analysis of the alpha diversity of the three enrichments revealed significant differences in the community structures among the three enrichments. The community structure of AT was much more complex and diverse than those of enrichments ATB and ATW. The results revealed that artificial interventions (such as successive enrichments) affect the bacterial community structure greatly, considering that enrichments ATB and ATW were derived from AT by incubated it with and without atrazine, respectively, for an additional 20 days. Similarly, the effects of atrazine on the structure of the soil microbial communities have been reported in many studies (Ghani et al., 1996; Sannino and Gianfreda, 2001; Moreno et al., 2007; Mahía et al., 2011). The alpha index between enrichments ATB and ATW was not obviously different, indicating that atrazine was not the only factor that changed the structure of the bacterial community (Wang et al., 2015). However, significant differences in the atrazine degradation efficiency were observed in these two enrichments, which might have resulted from their specific community compositions. For example, the relative abundance of *Arthrobacter* (the atrazine degrader) in enrichment ATB was approximately 20 times more than that in enrichment ATW, and it was also much higher than that in AT. In parallel to the 16S rRNA analysis, we performed independent isolation screens for atrazine degraders from the original enrichment AT, resulting in the functional-based identification of one isolate classified as *Arthrobacter* that was confirmed as an atrazine degrader. Degradation of atrazine by *Arthrobacter* species has also been extensively reported (Sajjaphan et al., 2004; Wang and Xie, 2012; Xie et al., 2013).

A previous study proposed that the relative abundance of atrazine-degrading bacteria, including *Arthrobacter*, increased in soils that were repeatedly treated with atrazine (Fang et al., 2015). However, few studies have focused on the alteration of other bacteria, especially the non-degraders. In this study, aside from *Arthrobacter*, two other genera, *Hyphomicrobium* and *Methylophilus*, showed a significant higher abundance in enrichment ATB than that in enrichments AT and ATW. The abundance of the two genera showed no correlation with the atrazine degradation rate but had a significantly positive co-occurrence with *Arthrobacter*, showing they could also improve the atrazine degradation rate and may indirectly participate in atrazine degradation by promoting the growth of *Arthrobacter*. This conclusion was supported by the results of *in vitro* experiments that showed that *Methylophilus* improved the atrazine degradation rate when co-inoculated with *Arthrobacter* after 48 h. The lag in the improvement after inoculation is consistent with the proposed indirect participation in atrazine degradation. These microbes might use the metabolites produced from atrazine degradation and cross-feed the *Arthrobacter* or maintain a stable community structure, promoting the degradation of atrazine (Piutti et al., 2002). Specifically, *Hyphomicrobium* has been mentioned in previous studies on the degradation of dichloromethane or methanol (Timmermans and Haute, 1983; Kohler-Staub and Leisinger, 1985).

Several bacteria, such as *Halomonas, Azospirillum, Dokdonella*, Planctomycetaceae, Rhodospirillaceae, and Phyllobacteriaceae, were found to suppress atrazine degradation, as their abundance showed a significant negative correlation with the atrazine degradation rate. All these species had the highest abundance in enrichment ATW. No co-occurrence of them with atrazine-degradation-promoting bacteria was found, indicating that these bacteria might suppress atrazine degradation directly instead of inhibiting the growth of other bacteria. The experimental results also support the conclusion that *Halomonas* and *Azospirillum* suppressed atrazine degradation immediately after co-inoculation with *Arthrobacter* without a lag phase. In addition to the genera that directly affected atrazine degradation, bacteria that indirectly inhibited atrazine degradation efficiency were also detected. Fifteen biomarkers in AT that had no correlation with the atrazine degradation rate were detected, but they had a significant negative co-occurrence with the potential atrazine-degradation-enhanced bacteria, revealing that these bacterial might affect the atrazine degradation rate via influencing the growth of the atrazine-degradation-enhanced bacteria.

Although the relative abundance of *Arthrobacter* in enrichment ATW was higher (6.17%) than that in enrichment AT (1.95%), a lower degrading efficiency of atrazine in enrichment ATW was detected. This phenomenon might result from the relatively high abundance of bacteria that suppressed atrazine degradation such as *Azospirillum*, which might inhibit the degradation activity of *Arthrobacter* strains. Furthermore, the same strain at different abundances might have different effects on atrazine degradation. For example, a higher abundance of *Halomonas* inhibited the atrazine-degrading efficiency, while a lower abundance of *Halomonas* improved the atrazine-degrading rate by *Arthrobacter.* In agreement with the experimental results, the sequencing data showed that enrichment ATW had a much higher abundance of *Halomonas* than that in enrichments AT and ATB, which may suppress atrazine degradation in enrichment ATW but promote degradation in enrichments AT and ATB. It has been shown that the presence of some bacteria might alter the proportion of some organic components (such as the C/N ratio) in the environment, thereby affecting the degradation of atrazine (Cook and Huetter, 1981; Alvey and Crowley, 1995; Abdelhafid et al., 2000). These results suggest that there are complicated and flexible interactions among members in consortia, not only caused by the different compositions but also derived from the different abundances. The shifts in these interactions in consortia drive the change in catabolic function efficiency.

In summary, the relationships between atrazine degrading efficiency and microbial community structures in three enrichments were explored. The bacterial community composition and structure significantly affected atrazine degrading efficiency. The efficiency of atrazine degradation was influenced by not only the key degraders but also some indirect species (non-degrader) in the community. Some species that might be beneficial or detrimental to the atrazine degraders were identified, providing useful information for the design of bioremediation strategies for contaminated soil.

## Author Contributions

KC and JJ conceived and designed the experiments. XL performed the experiments. XX and SC analyzed the data. XL and XX prepared the manuscript. JJ, XL, and XX revised the manuscript. All authors contributed to manuscript and read and approved the submitted version.

## Conflict of Interest Statement

The authors declare that the research was conducted in the absence of any commercial or financial relationships that could be construed as a potential conflict of interest.
