# Supplementary material for: Shift in Bacterial Community Structure Drives Different Atrazine-Degrading Efficiencies
Source: Front Microbiol. 2019 Jan 30;10:88. doi: 10.3389/fmicb.2019.00088 (PMC6363660; doi:10.3389/fmicb.2019.00088)
Supplement: Supplementary file 1 [file Table_1.DOCX]

Table S1. Barcodes of paired-end sequences of all samples subjected to multiplex pyrosequencing

| Sample ID | Fbarcode | Rbarcode |
| --- | --- | --- |
| AT1 | CCACAA | GCCAAT |
| AT2 | TCGAAG | AGTGGA |
| AT3 | ACAGTG | TTCAGA |
| ATB1 | GAATTCA | AACGCCT |
| ATB2 | ATGAAAC | GAACTTC |
| ATB3 | TATCGGGA | CCGGATAT |
| ATW1 | CATCGT | TTCAGA |
| ATW2 | GCTTA | ATTGA |
| ATW3 | ACGTGTT | GTCGATT |

Table S2. Sequences and modified OTUs of all samples from MiSeq pyrosequencing at 97% identity

| Sample ID | Valid sequences | High quality sequences | Average Length(bp) | Modified OTUs |
| --- | --- | --- | --- | --- |
| AT1 | 70,663 | 59,566 | 438.92 | 791 |
| AT2 | 76,892 | 66,712 | 438.63 | 791 |
| AT3 | 83,306 | 71,792 | 439.82 | 790 |
| ATB1 | 47,235 | 46,904 | 442.42 | 94 |
| ATB2 | 41,756 | 41,476 | 442.20 | 99 |
| ATB3 | 57,120 | 56,673 | 441.52 | 104 |
| ATW1 | 44,159 | 43,695 | 442.1 | 136 |
| ATW2 | 43,650 | 43,182 | 441.48 | 157 |
| ATW3 | 41,082 | 40,625 | 440.81 | 141 |
| Total | 505,863 | 470,625 | 440.57 | 867 |


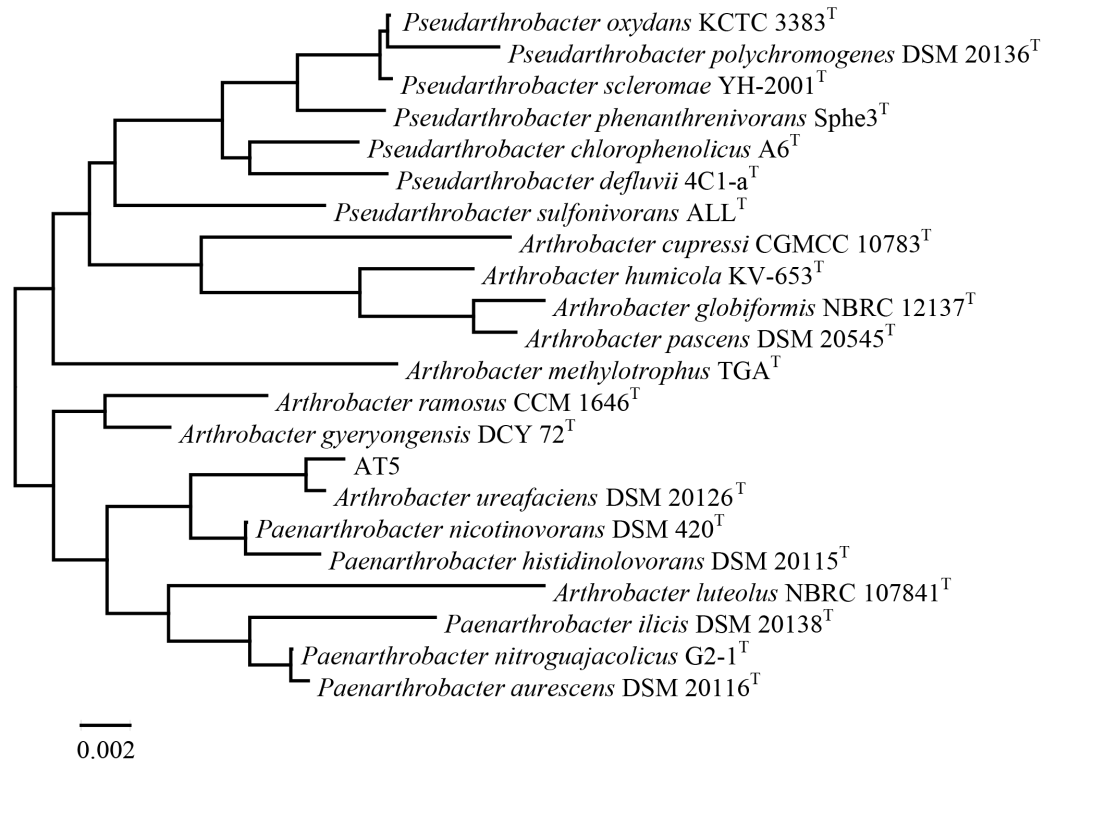


Figure S1. Phylogenetic relationship between Arthrobacter sp. strain AT5 and other closely related species based on 16S rRNA gene sequences


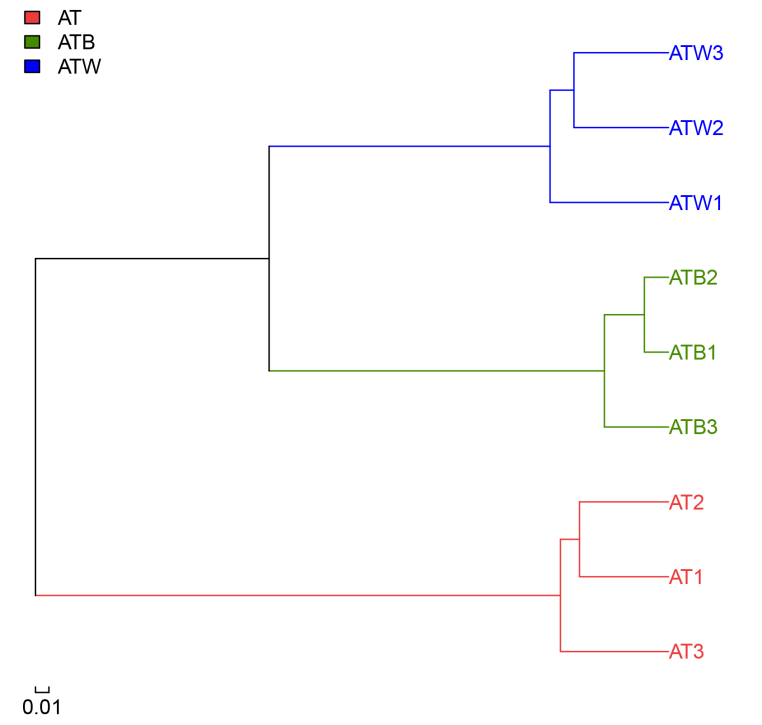


Figure S2. Hierarchical clustering with Bray–Curtis distances of bacterial communities.


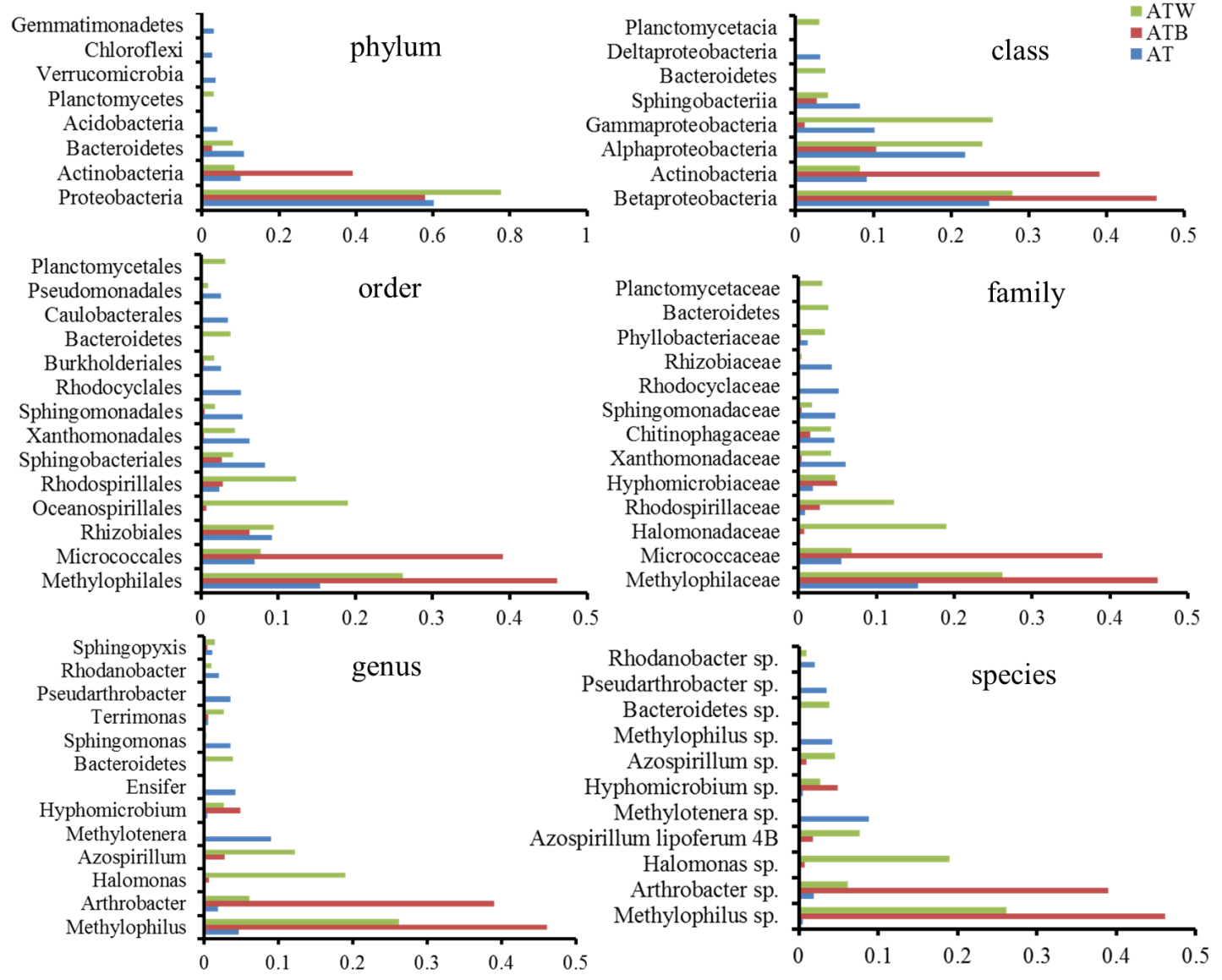


Figure S3. Distribution of the predominant bacteria at different taxonomic levels (phylum, class, order, family, genus, and species). The predominant taxa (>1% relative abundance) in each level are shown. Columns of different colors represent different groups: green represents group ATW, red represents group ATB, and blue represents group AT. The length of the column represents the relative abundance in the group.


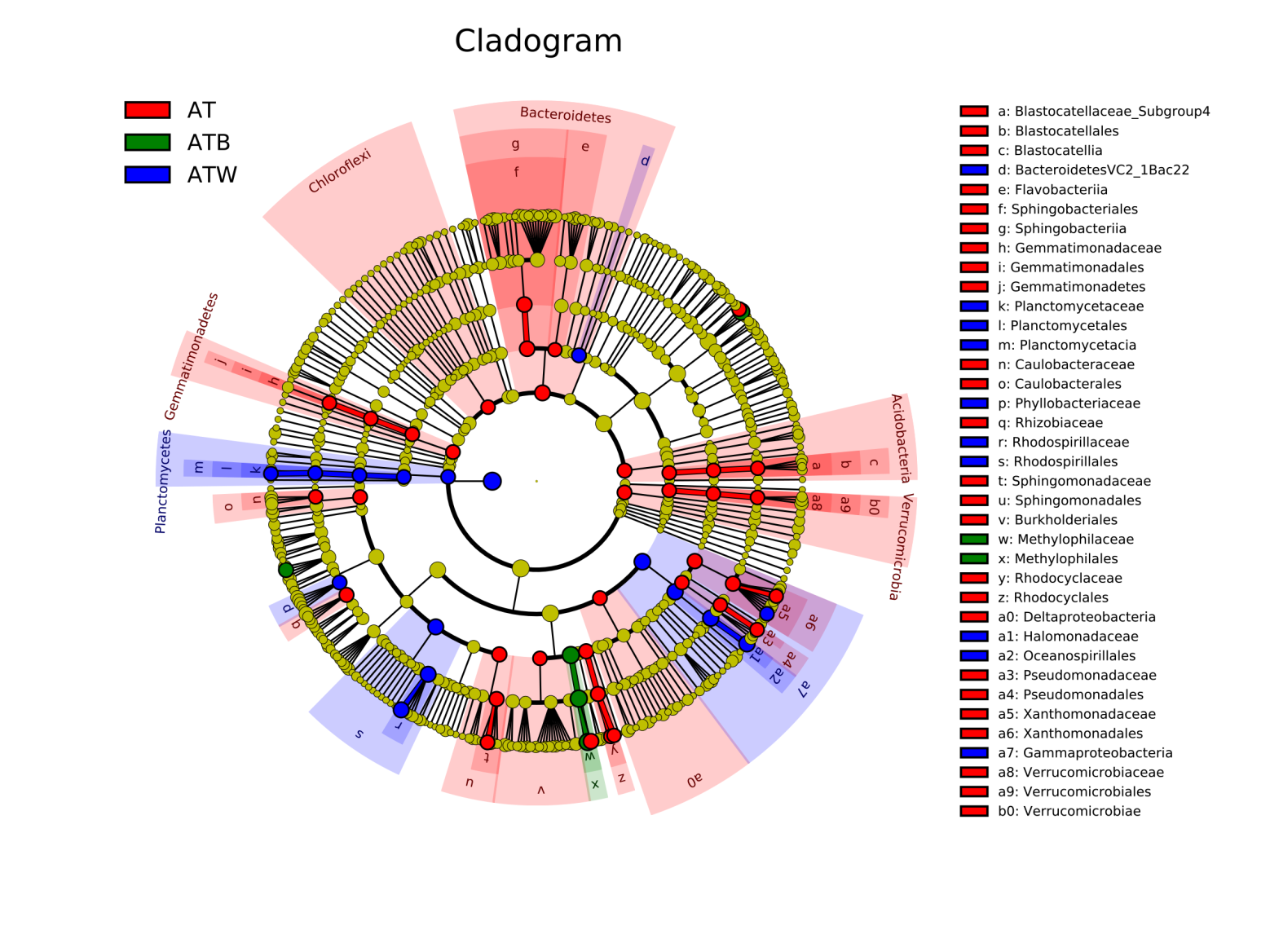
Figure S4. LEfSe analysis cladogram. The circles radiating from the inside to the outside of the evolutionary branching graph represent the classification level from the phylum to the species. Each small circle at the different classification levels represents a classification at this level, and the size of the small circle is proportional to the relative abundance. The principle of the coloring is that species with no significant differences are colored yellow, and the other species are colored according to the group with the highest abundance of the species. Different colors represent different groups, and nodes of different colors represent the microbial communities that play an important role in the group.


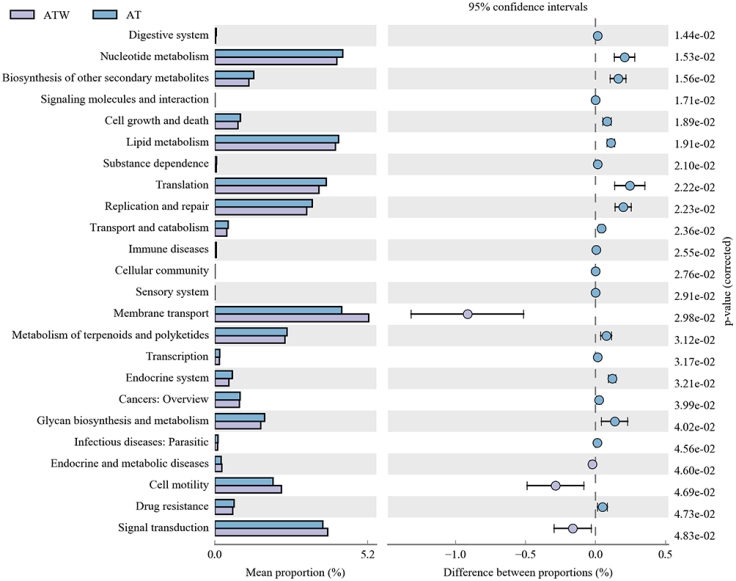


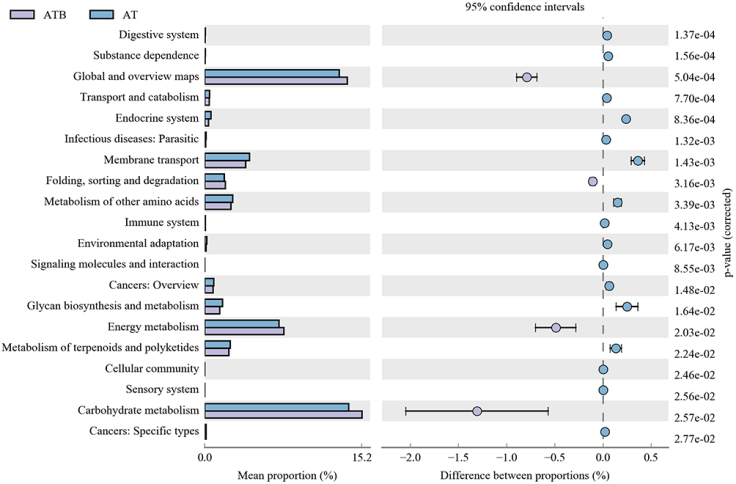


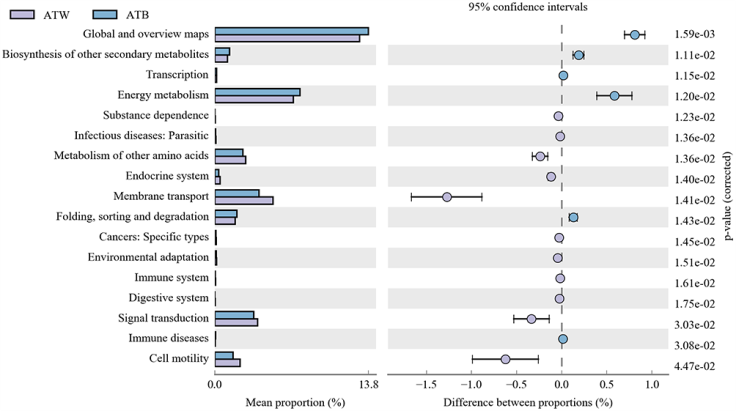


Figure S5. Analysis of metabolic pathway differences in KEGG terms between various combinations of two groups (A: ATW and AT, B: ATB and AT, C: ATW and ATB). In each diagram, the left figure shows the ratio of abundance of different functions in the two groups of samples, the middle shows the proportion of the differences in functional abundance in the 95% confidence range, and the right-most number is the p value.
